# Pheochromocytoma with Takotsubo Syndrome and acute heart failure: a case report

**DOI:** 10.1186/s12957-022-02704-0

**Published:** 2022-08-05

**Authors:** Lin Yang, Yiying Zhang, Yanqun Hu, Zhi Yang

**Affiliations:** 1 Department of Infectious Diseases, The Second Clinical Medical College of Jinan University (Shenzhen People’s Hospital), Shenzhen, China; 2 Department of Infectious Diseases, The 6th Affiliated Hospital of Guangdong Medical University (Shenzhen Nanshan People’s Hospital), Shenzhen, China; 3 Department of Endocrinology, Affiliated Baoan Hospital of Shenzhen, Southern Medical University (People’s Hospital of Baoan District), Shenzhen, China

**Keywords:** Pheochromocytoma, Takotsubo Syndrome, Acute heart failure, Catecholamine, Case report

## Abstract

**Background:**

Pheochromocytoma is a neuroendocrine tumor that can overproduce catecholamines. Heart failure and Takotsubo Syndrome (TTS) caused by excessive catecholamines are uncommon pheochromocytoma complications.

**Case presentation:**

A 27-year-old woman was referred to our center for further preoperative assessment and adrenalectomy. She came to the emergency ward with the typical symptoms of acute coronary syndrome and heart failure, including chest stuffiness, dyspnea, epigastric pain, and diaphoresis. The high level of 24-hour urinary vanillylmandelic acid and abdominal computed tomography findings supported the diagnosis of pheochromocytoma. Transthoracic echocardiography showed diffuse hypokinesis of the left ventricular wall with an ejection fraction of 23%. All symptoms and left ventricular function recovered rapidly after left laparoscopic adrenalectomy. Histopathology findings confirmed the diagnosis of pheochromocytoma. Based on the above findings, we eventually diagnosed her with pheochromocytoma-induced TTS.

**Conclusions:**

This is a rare case of pheochromocytoma without hypertension complicated by TTS and acute heart failure. A diagnosis of pheochromocytoma-induced TTS should be considered for patients presenting with uncommon heart failure, even in patients without hypertension. Standard treatment is the surgical removal of the adrenal mass.

**Supplementary Information:**

The online version contains supplementary material available at 10.1186/s12957-022-02704-0.

## Background

Pheochromocytoma is an uncommon and catecholamine-producing tumor originating from the adrenal medulla and is often complicated with headache, palpitation, polyhidrosis, and hypertension [1, 2]. Heart failure and Takotsubo Syndrome (TTS) are rare and life-threatening cardiovascular complications of pheochromocytoma [3, 4]. Therefore, these rare cardiovascular manifestations as the first symptoms of pheochromocytoma often lead to a delay in diagnosis. The timing of surgery in patients with pheochromocytoma-induced TTS and acute heart failure is controversial. In this report, we show a case of a patient with acute heart failure and TTS induced by pheochromocytoma. In addition, the patient presented with hypotension. After intensive medical stabilization and left adrenal tumor resection, blood pressure was normalized and heart failure was relieved.

## Case presentation

A 27-year-old woman without prior medical history came to the emergency ward with dyspnea, epigastric pain, diarrhea, chest stuffiness, headache, emesis, and diaphoresis. All of these symptoms except dyspnea had been ongoing for a month and the patient was treated with antispasmodic and proton-pump inhibitors at a local hospital before admission. She complained of mild dyspnea after daily activities for half a month, which gradually developed into paroxysmal nocturnal dyspnea. The patient had a history of cesarean delivery six years ago. She disclaimed any family history of pulmonary or cardiovascular diseases or malignant tumors.

**Fig. 1:**
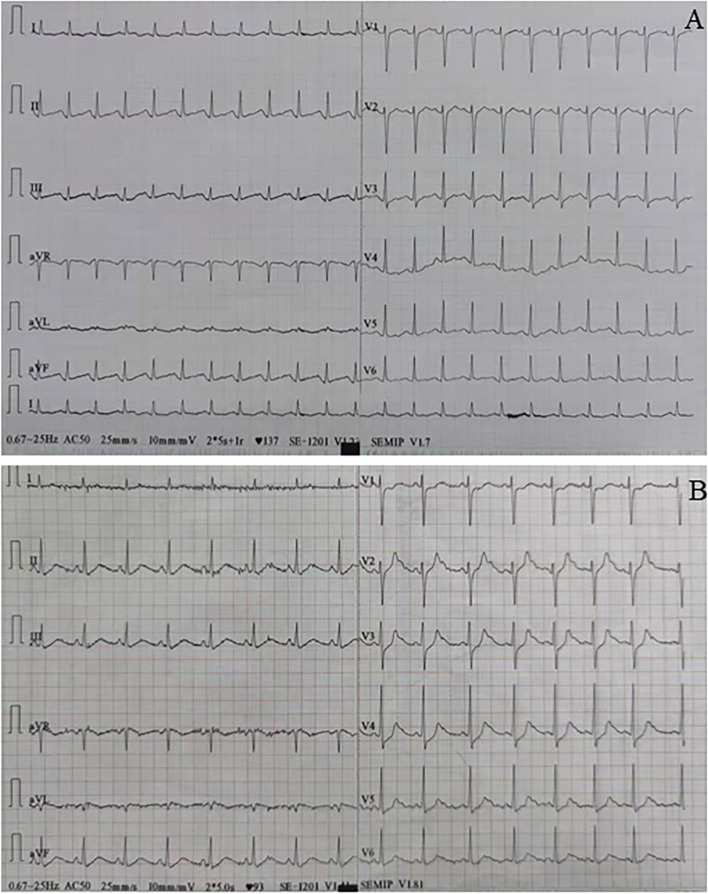
**A** Electrocardiograph showed sinus tachycardia. **B** Electrocardiograph showed ST-segment depression in II, III, AVF, V3, and V4 tracings

**Fig. 2:**
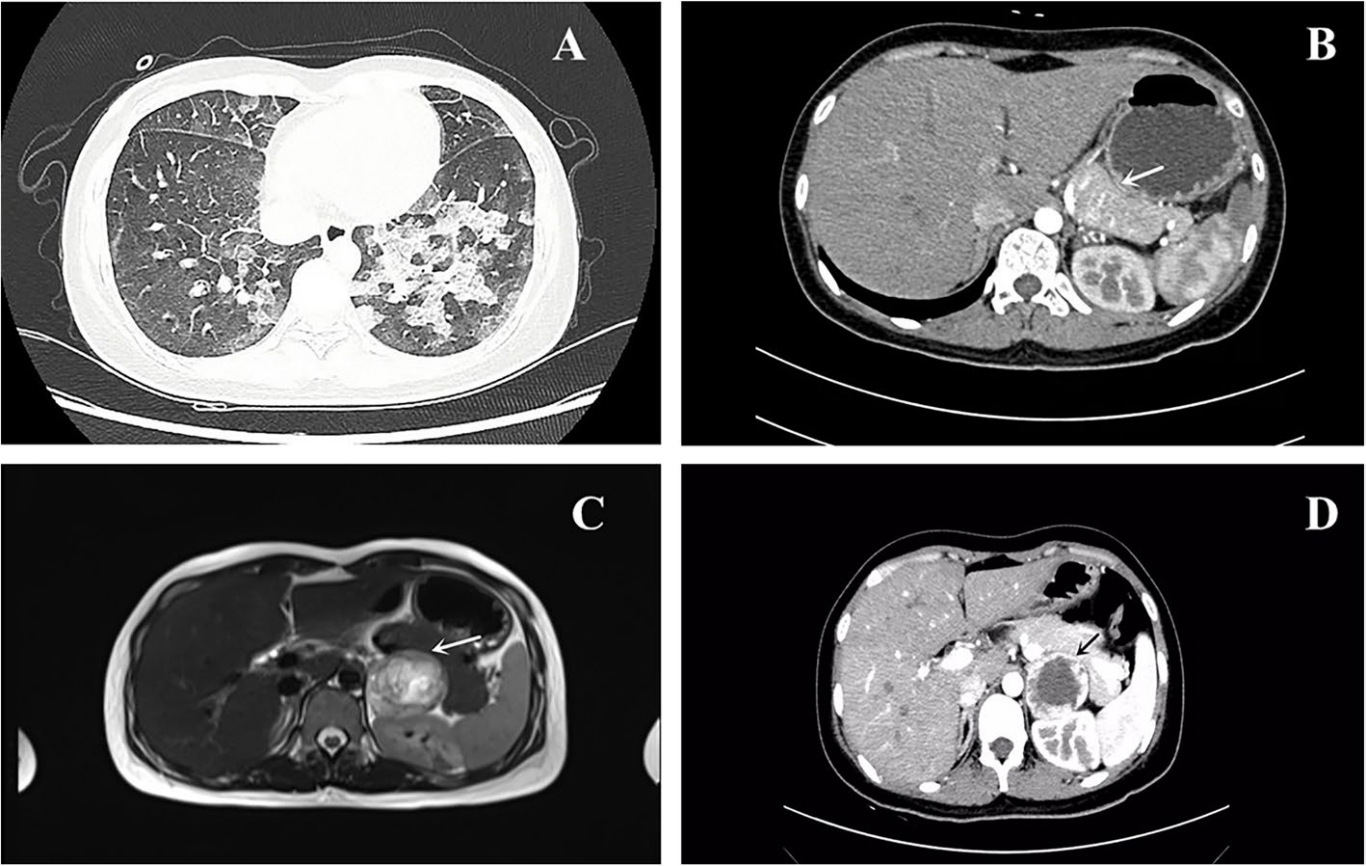
**A** Chest computed tomography scan showing bilateral pulmonary edema. **B** Abdominal CT scan: a left adrenal mass measuring 4 × 5 cm. **C** Adrenal MRI: a left adrenal tumor measuring 5.6 × 4.6 cm with hemorrhage. D Enhanced adrenal CT showing a left adrenal mass with an uneven density measuring 4.8 × 4.1 cm

At presentation, the physical examination revealed a blood pressure of 88/65 mmHg, a respiratory rate of 23 breaths per minute, and a pulse of 145 beats per minute. Lung auscultation revealed diffuse moist rales in the lungs. An abdominal examination showed epigastric tenderness. The remainder of the examination results were unremarkable. During her stay in the emergency ward, the patient was treated with phloroglucinol, promethazine, and an injection of saline for gastrointestinal symptoms. She later displayed orthopnea, profuse sweating, severe coughing with pink frothy sputum, and a drop in blood pressure (80/55 mmHg). Table 1 shows significant laboratory test results on admission. The arterial blood gas (ABG) was potential of hydrogen (PH) 7.46, arterial partial pressure of oxygen (PaO_2_) 72.80 mmHg, and base excess (BE) -6.1 mmol/L. The N terminal pro B type natriuretic peptide (NT-ProBNP) was 1720.0 pg/ml (less than 300 pg/ml can rule out heart failure). Other laboratory findings showed that the white blood cell count (WBC) was 18.2×10^9^/L (reference range: 4−10×10^9^/L), the neutrophil ratio was 87.4% (reference range: 50-75%), creatine kinase MB (CK-MB) was 20.1 ng/ml (reference range: 0−3.10 ng/ml), troponin I 8.1 ng/ml (reference range: 0-0.034 ng/ml), myoglobin (MYO) was 132.6 ng/ml (reference range: 3.5-22.8 ng/ml). Electrocardiograms (ECGs) on admission suggested sinus tachycardia (Fig. 1A) and ischaemic changes (ST-segment depression in II, III, AVF, V3, and V4 tracings) (Fig. 1B). A chest computed tomography (CT) scan (Fig. 2A) showed bilateral pulmonary edema and an abdominal CT scan (Fig. 2B) revealed a left adrenal mass measuring 4 × 5 cm.

**Table 1:** Initial laboratory findings of the patient

Blood routine examination	Results	Reference range
WBC	18.2×10^9^ /L	4-10×10^9^ /L
Neutrophil ratio	87.4 %	50-75 %
Myocardial enzyme		
CK-MB	20.1 ng/ml	0-3.10 ng/ml
Troponin I	8.1 ng/ml	0-0.034 ng/ml
MYO	132.6 ng/ml	3.5-22.8 ng/ml
NT-ProBNP	1720.0 pg/ml	< 300 pg/ml
Endocrinology markers		
3-methoxytyramine	0.11 noml/L	≤ 0.18 nmol/L
Metanephrine	1.04 noml/L	≤ 0.50 noml/L
Normetanephrine	20.56 nmol/L	≤ 0.90 noml/L
Urinalysis		
24 h urine VMA (mg/24 h)	48.51 mg/24h	0-12 mg/24h

*WBC* white blood cell count, *CK-MB* creatine kinase MB, *MYO* myoglobin, *NT-ProBNP* N terminal pro B type natriuretic peptide; 24 h urine VMA, 24-hour urinary vanillylmandelic acid

Considering all the symptoms and the chest and abdominal CT results, the diagnosis was suspected to be acute heart failure, pheochromocytoma, and catecholamine cardiomyopathy. The patient was placed in Coronary Care Unit (CCU). Non-invasive ventilator-assisted ventilation was given. Torasemide, Recombinant Human Brain Natriuretic Peptide, and digoxin were given to correct heart failure. After the above treatment, the patient’s symptoms improved, and the levels of Troponin I, MYO, CK-MB, and NT-ProBNP gradually recovered normally. Plasma metanephrines (3 items) showed that 3-methoxytyramine was 0.11 noml/L (reference range: ≤ 0.18 nmol/L), metanephrine was 1.04 noml/L (reference range: ≤ 0.50 noml/L), and normetanephrine was 20.56 nmol/L (reference range: ≤ 0.90noml/L). 24-hour urinary vanillylmandelic acid (VMA) was 48.51 mg/24h (reference range: 0-12 mg/24h). Transthoracic echocardiography (TTE) showed diffuse hypokinesis of the left ventricular wall with a left ventricle ejection fraction (LVEF) of 23%. On day 7 of admission, reexamination of TTE shows a reduction of abnormal systole and an improvement of LVEF, with recovery to 50%. Bedside chest radiography showed that the lesions of pulmonary were markedly improved. Subsequently, adrenal magnetic resonance imaging (MRI) and cardiac MRI were performed. Adrenal MRI reveals a left adrenal tumor measuring 5.6 × 4.6 cm with hemorrhage (Fig. 2C). There was no evidence of extra-adrenal lesions, so we recommended left adrenalectomy after sufficient preoperative preparation. Doxazosin was prescribed to provide a sufficient alpha-blocker before the operation. She was transferred to our center in a relatively stable condition.

The patient was admitted to the endocrinology department for adequate preoperative preparation, which included BP control with phenoxybenzamine and metoprolol and an injection of normal saline for preventing severe hypotension after tumor resection. During the period of preoperative preparation, and an enhanced adrenal CT showed a left adrenal mass with an uneven density measuring 4.8 × 4.1 cm (Fig. 2D). Whole-exome sequencing genetic testing reported that no genetic mutations associated with pheochromocytoma were detected.

**Fig. 3:**
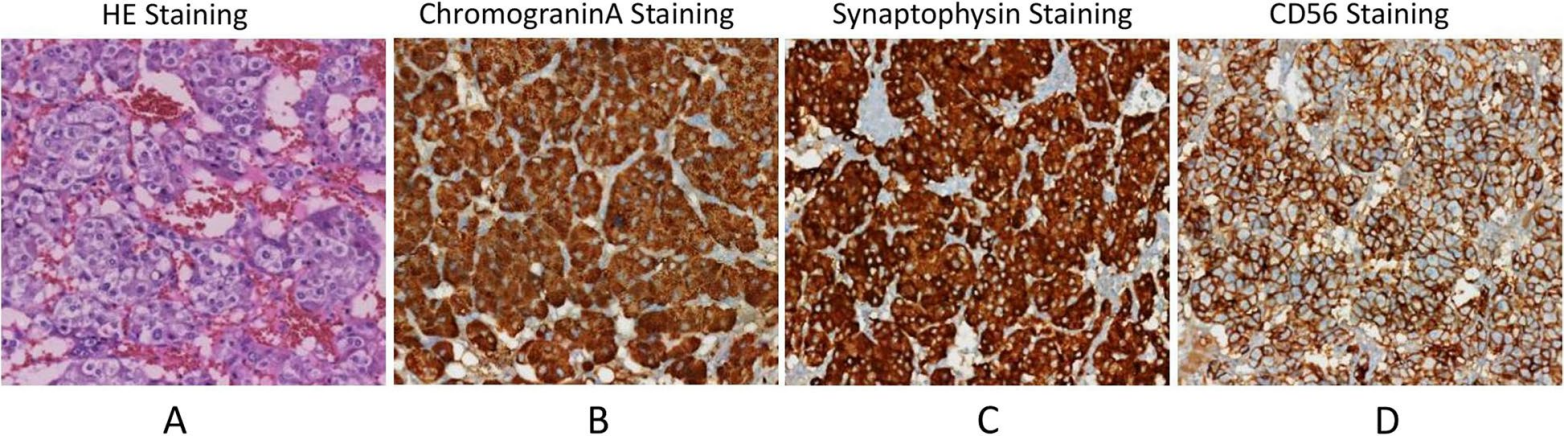
**A** tumor cells in a nested arrangement and surrounded by fibrovascular stroma; **B** tumor cells with basophilic glandular cytoplasm without atypia; chromogranin A positivity; **C** synaptophysin positivity; **D** CD56 positivity. Chromogranin A, synaptophysin and CD56 are neuroendocrine markers

Three weeks after her referral, laparoscopic adrenalectomy was successfully performed with the assistance of the da Vinci Surgical System. The report of pathological examination confirmed a pheochromocytoma with hemorrhage and necrosis (Fig. 3). Immunohistochemistry showed Syn (+), CD56 (+), CgA (+), S-100 (+), α-inhibin (-), HMB45 (-), Melanie (-), Ki-67 (5% +). In immunostaining of the resected adrenal tumor, focal necrosis was observed. Therefore, pheochromocytoma of adrenal gland scoring scale (PASS) was 2 points. Seven days after surgery, she was discharged from the hospital in good condition. During five months of regular follow-up, her BP and heart rate normalized, and she had no dyspnea, headache, emesis, or abdominal pain. Her levels of the myocardial enzyme, plasma metanephrine, 24-hour urine VMA, calcitonin, and parathyroid hormone were within the normal range. The findings of 24-hour ambulatory blood pressure monitoring, ECG, and TTE were normal. Furthermore, adrenal CT did not reveal new lesions. Although the pathological examination of the tumor was benign and the genetic test showed no mutation, we recommended that she come to the hospital for a follow-up survey regularly.

### Review of the Literature

We performed a literature search and identified 87 pheochromocytoma cases with Takotsubo Syndrome or cardiomyopathy. Of the 87 cases, 56 patients were selected from a review [5]. Presentation and outcome of the 88 cases (including our case) are summarized in Table 2, and detailed clinical descriptions and references are available in Supplemental Table 1.

**Table 2:** Clinical characteristics of pheochromocytoma-with Takostubo syndrome or cardiomyopathy

*N*=88 cases		
Sex	F: 62 (71%)	M: 25 (29%)
Age (mean)	F: 42 (21-81)	M: 46 (23-86)
Trigger	17 (19%)	
Clinical manifestation		
CP	47 (53%)	
Classic symptoms	Headache (20.4%)	
	Palpitation (15.9%)	
	Diaphoresis (20.4%)	
BP	High 33 (37.5%)	Low 7 (8.0%)
ECG	82 (93.2%)	
ST elevation/depression	55 (67.1%)	
Initial echocardiograpy	53 (60.2%)	
Myocardial infraction markers	70 (79.5%)	
Increase in Troponin	68 (97.1%)	
Coronary angiography	78 (88.6%)	
No CAD	70 (89.7%)	
Genetic Mutation	8 (9.1%)	
Recurrence of cardiac event after excision	0	
Emergency adrenalectomy	8 (9.1%)	
Death (Y/N)	6 (6.8%)	

*M* Male, *F* Female, *CP* Chest pain

*M* Metanephrine, *NM* Normetanephrine, *E* epinephrine, *NE* norepinephrine

*MIBG* Metaiodobenzylguanidine

*ECG* Electrocardiogram

*LVEF* Left ventricular ejection fraction, CAD: Coronary artery disease

Of the 88 cases, sixty-two (71%) were women, and the mean age was 42 years, ranging from 21 to 81 years. One of the patients was a pregnant woman. Twenty-five (29%) were men and mean age was 46 years, ranging from 23 to 86 years. A trigger event was difficult to identify in most patients. The most common initial presentation of patients is chest pain in 47 patients (53%), similar to that of acute myocardial infarction. Other classic presentations pheochromocytoma including, headache (20.4%), palpitation (15.9%), diaphoresis (20.4%) and hypertension (52.6%). Initial low blood pressure was reported in 7 patients including our case.

Information about cardiac biomarkers was available in 70 (79.5%) cases, and 68 (97.1%) had increased troponin. Initial echocardiography was completed in 53 (60%) cases. ECG was reported in 82 (93.2%) cases. The most common ECG changes were ST elevation or ST depression. Coronary angiography was completed in 78 (88.6%) patients, showing no coronary disease in 70 (89.7%) cases and minor disease in 8 patients. Genetics tests were only completed in 8 cases (9.1%). Complications were described in 50 patients (56.8%). Cardiogenic shock and heart failure were reported in 26% and 42% of them respectively. Five patients (5.7%) required the support of extracorporeal membrane oxygenation (ECMO). The prognosis of most patients was good after adrenalectomy. There were no recurrent cardiovascular events were reported. The mortality rate amongst the patients on admission was 6.8%.

## Discussion

Pheochromocytoma is a neuroendocrine tumor characterized by headache, sweating, and palpitations [6]. Due to the different forms and amounts of catecholamines secreted by tumors, the clinical features of pheochromocytoma are varied and difficult to identify. Some patients were even asymptomatic. Some patients came to the hospital because of the rare complications of pheochromocytoma, which can easily lead to misdiagnosis and missed diagnosis clinically.

Here, we analyzed the cases reported in previous literatures along with our case for a total of 88 patients. Our review revealed that women were at higher risk of developing pheochromocytoma-induced TTS than men. A trigger was identified in about one fifth of cases. The most frequent trigger was surgery (6 cases). The patients were not always presenting with the classical triad of pheochromocytoma symptoms. Recurrence of TTS occurred in 14 out of 88 (16%) cases as similarly found by previous literature that reported recurrence of TTS in 9 out of 49 individuals (18.4%) with pheochromocytoma and paraganglioma [7]. Of note, in our analysis, no recurrence of TTS in all cases occurred after adrenalectomy. Malignant pheochromocytoma was reported in only one case which was presented by Kaneto et al. [8]. This report showed a Japanese male who was finally diagnosed as malignant pheochromocytoma accompanied by bone and multiple liver metastases and TTS. Moreover, there was no recurrence for 2 years after the operation, and LVEF was increased up to normal. It has been recommended that all patients with pheochromocytoma should be considered for genic test as one third of pheochromocytoma patients have disease-causing gene mutation [9, 10]. In addition, the mutations of succinate dehydrogenase B (SDHB) may lead to metastatic disease in at least 40% affected patients [10]. However, we found gene detection was performed in only 8 cases (9.1%) in our analysis. There can be several reasons. In clinical practice, patients may reject genetic testing due to the high cost. Moreover, some clinicians may lack awareness of the importance of genetic testing.

TTS and acute heart failure are both rare presenting features of pheochromocytoma [3, 11]. TTS was first presented by stao et al [12] in 1990, and it is an uncommon cardiomyopathy with clinical manifestations and ECG similar to acute myocardial infarction, but without coronary stenosis or spasm [13, 14]. Takotsubo syndrome takes its name from the Japanese word for octopus trap due to the shape of the LV at end-systole. The pathophysiology mechanisms of TTS were diverse and complex. Oxidation products of excessive catecholamines can produce a direct toxic effect on the myocardium by increasing sarcolemmal permeability and cellular calcium influx [15]. Moreover, catecholamine excess may lead to a switch from Gs to Gi protein via β2 adrenoreceptor, resulting in an impairment of cardiac contractility [16]. Other cardiac complications include hypertrophic cardiomyopathy, dilated cardiomyopathy, myocardial ischemia, and arrhythmias [3, 17].

TTS has got a prevalence of around 4-18% among patients with pheochromocytoma and paraganglioma [18]. However, plenty of patients were diagnosed with pheochromocytoma until cardiac events occurred. TTS is characterized by a transient left ventricular wall motion abnormality (LVWMA) and shares common clinical manifestations with acute coronary syndromes (ACS), such as chest pain, elevated myocardial enzyme, and ECG abnormalities. Based on the available data, women are at a higher risk of developing TTS, especially those older than 55 years [19].

In this case, our patient presented with typical symptoms of ACS, transient left ventricular dysfunction, ST-segment depression, and significant elevation of elevated myocardial enzyme and NT-ProBNP. Based on the International Takotsubo Diagnostic Criteria [19], the diagnosis of TTS was confirmed. Histopathology examination and rapid LVWMA recovery supported the diagnosis of pheochromocytoma-induced TTS and the deduction that acute heart failure was caused by TTS. It is worth mentioning that the patient was persistently hypotensive until heart failure was corrected. It is commonly reported that at least 80% of patients with pheochromocytoma have persistent or paroxysmal hypertension [20]. For our patient, there were several possible causes for the hypotension. First, low cardiac output due to heart failure might contribute to the drop in BP in this patient. The sensitivity of catecholamine receptors might be suppressed by the long-term and excessive elevation of catecholamine levels. Third, some inactive catecholamine metabolites in the tumor are secreted into the blood. Therefore, a diagnosis of pheochromocytoma should be suspected when heart failure is unexplainable and especially when accompanied by significant abdominal pain, hypotension, profuse sweating, and leukocytosis. When a patient is diagnosed with pheochromocytoma, genetic testing is recommended. About 15-20% of patients with pheochromocytoma are metastatic, and some of them were confirmed to have metastasized during the follow-up period [21, 22]. Approximately 40% of patients with pheochromocytoma and paragangliomas are associated with gerne mutations, which makes pheochromocytoma solid tumors with a high heritability rate [23]. The major mutations associated with pheochromocytoma are von Hippel-Lindau gene (VHL), REarranged during Transfection (RET) proto-oncogene, neurofibromatosis type 1 gene (NF1), genes encoding four succinate dehydrogenase complex subunits (SDHx; i.e., SDHA, SDHB, SDHC, and SDHD genes) [24]. Histopathologic grading of pheochromocytoma is usually assessed with the PASS [25]. In our case, the whole-exome sequencing genetic testing was negative and PASS was less than 4 points, indicating that the risk of metastasis in this subject was low [25]. Hence, the standard treatment is the surgical removal of the adrenal mass. Our patient recovered quickly after elective surgery. With regard to the optimal surgical time for pheochromocytoma patients with TTS, it is controversial. Some studies reported that delayed surgery seemed to be superior to emergency surgery unless the patient presented with shock due to rupture or hemorrhagic necrosis of a pheochromocytoma [26–28]. Generally, the prognosis of pheochromocytoma patients with TTS is relatively good after surgical resection.

There are disadvantages to this report. First, cranial MRI was not performed to confirm the presence of brain metastases. According to the fourth edition of the World Health Organization (WHO) classification of endocrine tumors, all pheochromocytomas are considered to have the potential to metastasize [29]. Second, the data we analyzed were from previous reported cases so that some details were not able to systematically obtained. In addition, coronary angiography was not performed to rule out coronary stenosis. It has been reported that ACS may co-exist with TTS [30], and ACS itself may be a trigger for TTS [31].

### Conclusion

We report a rare case of pheochromocytoma without hypertension complicated by heart failure and TTS. Our findings indicate that early diagnosis and proper therapy are significant since the LVWMA of patients with TTS may recover quickly after surgical resection. Moreover, a differential diagnosis of pheochromocytoma-induced TTS should be considered for patients presenting with uncommon heart failure, even in patients without hypertension.

## Supplementary Information

**Additional file 1.**

## Data Availability

All datasets presented in this study are included in the article. Further inquiries can be directed to the corresponding author.

## Abbreviations

TTS: Takotsubo Syndrome
ABG: The arterial blood gas
PH: the potential of hydrogen
PaO2: arterial partial pressure of oxygen
BE: base excess
NT-ProBNP: N-terminal pro brain natriuretic peptide
WBC: the white blood cell count
CK-MB: creatine kinase MB
MYO: myoglobin
ECG: Electrocardiogram
CT: computed tomography
VMA: vanillylmandelic acid
TTE: Transthoracic echocardiography
LVEF: left ventricle ejection fraction
MRI: magnetic resonance imaging
LVWMA: left ventricular wall motion abnormality.
