# Supplementary material for: Pheochromocytoma with Takotsubo Syndrome and acute heart failure: a case report
Source: World J Surg Oncol. 2022 Aug 5;20:251. doi: 10.1186/s12957-022-02704-0 (PMC9354339; doi:10.1186/s12957-022-02704-0)
Supplement: Supplementary file 1 — Additional file 1. [file 12957_2022_2704_MOESM1_ESM.docx]

**Supplementary Table 1** **Detailed clinical descriptions of cases report with Takostubo syndrome or cardiomyopathy (1-31)**

N=32 Patients (M: 6 F: 26)

| Author  year | Contadini, D.  2017 | Gravina, M  2017 | Iwase, J  2017 | Kudaiberdiev, T  2017 | Schmidt, K. H  2017 |
| --- | --- | --- | --- | --- | --- |
| Sex/Age | F/42 | M/23  (heavy smoker) | F/24 (pregnant)  (heavy smoker) | M/61 | F/25 |
| Trigger | - | - | - | Overwork | Exercise |
| [Clinical](javascript:;) [manifestation](javascript:;) | Headache | CP, nausea, vomiting, sweating,  dyspnea | severe dyspnea | Fatigue and nausea，  breathless | CP, weakness, nausea, vomiting, and headache |
| Blood pressure | High | Normal | Normal | High | - |
| Urinary Catecholamine  ULN | Elevated  (No values) | E 20x  NE 20.5x  M 1.1x  NM 333x | E 12x  NE 1.6x  M 2.7x  NM 0.7x | - | Elevated  (no normal range) |
| Plasma M and NM (ULN) | - | - | - | E 259x  NE 56x  D 128x | - |
| Increase in Troponin  (Y/N) | Y | Y | - | Y | Y |
| Initial echocardiograpy (%) | 10 | 40 | 5 | - | 25 |
| ECG | Sinus tachycardia,  T wave inversion | Sinus rhythm | - | - | Sinus tachycardia, T-wave inversion |
| Adrenal Scan or MRI | R adrenal mass | R adrenal mass of 5.9cm | L adrenal mass of 7.6 x 6.8 x 8.0 cm | - | L adrenal mass of 4.7x4.2 cm |
| MIBG Scintigraphy Uptake (Y/N) | Y | - | - | - | Y |
| Coronary angiography | No CAD | No CAD | - | - | No CAD |
| Genetic Mutation | - | - | - | - | - |
| Recurrence | - | N | - | - | N |
| Emergency adrenalectomy (Y/N) | N | N | Y | N | N |
| Cardiogenic pulmonary edema | N | N | Y | Y | N |
| Mechanical ventilation  (Y/N) | N | N | Y | N | N |
| ECMO (Y/N) | N | N | N | N | N |
| Death (Y/N) | N | N | N | Y | N |

| Author  year | Butt, K  2018 | Demea, A. D  2018 | Tafreshi, S  2018 | Takeshita, Y  2018 | Afana, M  2019 |
| --- | --- | --- | --- | --- | --- |
| Sex/Age | F/42 | F/52 | F/26 | M/46 | F/66 |
| Trigger | - | - | - | - | - |
| [Clinical](javascript:;) [manifestation](javascript:;) | Palpitations, flushing, headache, numbness, breathless | CP, dyspnea,  diaphoresis | Headache,  nausea, vomiting,  CP | Abdominal pain | Nausea,  epigastric pain |
| Blood pressure | High | Normal | High | Normal | High |
| Urinary Catecholamine  ULN | - | M 39x | Elevated  (no normal range) | E 1.2x  NE 1.3x  M 0.3x  NM 0.13x | E 3,3x  NE 1.8x  M 23x  NM 36x |
| Plasma Catecholamine (ULN) | - | - | - | Elevated  (no normal range) | - |
| Increase in Troponin  (Y/N) | - | Y | Y | Y | Y |
| Initial,echocardiograpy (%) | 25 | 40 | 38 | - | 50 |
| ECG | QT prolongation | ST elevation | ST depression | ST depression | ST elevation |
| Adrenal Scan or MRI | R adrenal mass of 4.4 x 3.3 cm | L adrenal mass of 12.4x10.4x10.0 cm | L adrenal mass of 12.5x6.3x6cm | L adrenal mass of 6.4x3.3cm | R adrenal mass of 11cm |
| MIBG Scintigraphy Uptake (Y/N) | - | - | - | Y | - |
| Coronary angiography | No CAD | No CAD | No CAD | No CAD | No CAD |
| Genetic Mutation | - | - | - | - | Negative |
| Recurrence | N | N |  | N | N |
| Emergency adrenalectomy (Y/N) | N | N | N | N (rupture and vanishing) | N |
| Cardiogenic pulmonary edema | N | N | N | N | N |
| Mechanical ventilation  (Y/N) | N | N | Y | N | N |
| ECMO (Y/N) | N | N | N | N | N |
| Death (Y/N) | N | N | N | N | N |

| Author  year | Diaz, B  2019 | Garla, V. V  2019 | Kiamanesh, O  2019 | Mierke, J  2019 | Chen, M  2020 |
| --- | --- | --- | --- | --- | --- |
| Sex/Age | F/50 | F/55 | F/45 | M/47 | M/70 |
| Trigger | Mental stress | - | - | Surgery | - |
| [Clinical](javascript:;) [manifestation](javascript:;) | CP | Dyspnea | CP, dyspnea, and headache | Serious hypotension | CP |
| Blood pressure | High | High | Low | Low | High |
| Urinary Catecholamine  ULN | - | M 11.7x | E 109x  NE 24x  M 51x  NM 8x | - | E 7x  NE 5x |
| Plasma Catecholamine (ULN) | - | M 4.6x  NM 8x | - | Elevated  (no values) | M 71x  NM 83x |
| Increase in Troponin  (Y/N) | Y | Y | Y | - | Y |
| Initial,echocardiograpy (%) | 15 | 10 | 15 | 15 | 34 |
| ECG | ST elevation | ST elevation | ST depression | - | ST depression |
| Adrenal Scan or MRI | R adrenal mass of 14cm | R adrenal mass of 4cm | R adrenal mass of 4.2x3.8 x 3.6 cm | L adrenal mass of 6.7cm | L adrenal mass of 9.6x8.3 cm |
| MIBG Scintigraphy Uptake (Y/N) | - | - | - | Y | N |
| Coronary angiography | No CAD | No CAD | No CAD | - | No CAD |
| Genetic Mutation | - | Negative | - | - | Negative |
| Recurrence | N | N | N | - | N |
| Emergency adrenalectomy (Y/N) | N | N | N | N | N |
| Cardiogenic pulmonary edema (Y/N) | N | - | Y | Y | N |
| Mechanical ventilation  (Y/N) | N | Y | Y | N | N |
| ECMO (Y/N) | N | N | Y | Y | N |
| Death (Y/N) | N | N | N | N | N |

| Author  year | Sato, K  2020 | Sethi, P  2020 | Spapen, J  2020 | Pierpaolo F  2020 | Sakul, N. F. N  2020 |
| --- | --- | --- | --- | --- | --- |
| Sex/Age | F/50 | F/51 | F/43 | F/69 | F/23 |
| Trigger | - | - | Hiking | - | - |
| [Clinical](javascript:;) [manifestation](javascript:;) | CP,dyspnoea, | Palpitations, headache,  nausea, vomiting | Nausea,  CP dyspnoea | CP,  tachycardia, diaphoresis | Nausea, vomiting,  CP |
| Blood pressure | - | High | High | High | Normal |
| Urinary Catecholamine  ULN | M 0.78x  NM 1.8x | M14X | M 31x  NM 15x | M 11x  NM 4x | - |
| Plasma Catecholamine (ULN) | - | M 10x | - |  | M 148x  NM 151x |
| Increase in Troponin  (Y/N) | - | Y | Y | Y | Y |
| Initial,echocardiograpy (%) | - | 55 | 35 | 35 | - |
| ECG | Normal | Peaked T waves | 1. wave progression,   QT prolongation | ST elevation  ST depression | Sinus tachycardia with a right bundle branch  block |
| Adrenal Scan or MRI | R adrenal mass of 3.5x3.5cm | L adrenal mass of 6cm | L adrenal mass of 59x 56 mm | R adrenal mass of 5 × 5.3cm | R adrenal mass |
| MIBG Scintigraphy Uptake (Y/N) | - | - | - | - | - |
| Coronary angiography | No CAD | No CAD | No CAD | - | No CAD |
| Genetic Mutation | - | - | - | Negative | - |
| Recurrence (Y/N) | Y | Y | N | N | N |
| Emergency adrenalectomy (Y/N) | Y | N | N | N | N |
| Cardiogenic pulmonary edema (Y/N) | Y | N | Y | N | N |
| Mechanical ventilation  (Y/N) | N | N | N | N | Y |
| ECMO (Y/N) | N | N | N | N | Y |
| Death (Y/N) | N | N | N | N | Y |

| Author  year | Jiang, X  2021 | Maffè, S  2021 | Marino, G  2021 | Martínez, A.  2021 |
| --- | --- | --- | --- | --- |
| Sex/Age | F/63 | M/53 | M/69 (smoker) | F/21 |
| Trigger | - | Fall | - | - |
| [Clinical](javascript:;) [manifestation](javascript:;) | Dyspnea,  cough with frothy pink  sputum,  sweating | Back and retrosternal pain | CP, sweating | CP, diaphoresis,  shortness of breath |
| Blood pressure | High | High | High | High |
| Urinary Catecholamine  ULN | - | - | M 46x  NM 12x | M 58x  NM 44x |
| Plasma Catecholamine (ULN) | M 17x  NM 61X | - | - | - |
| Increase in Troponin  (Y/N) | Y | Y | Y | Y |
| Initial echocardiograpy (%) | 38 | 20 | 23 | 40 |
| ECG | Premature atrial contractions | Sinus tachycardia with left bundle branch block | T waves inversion | T waves inversion |
| Adrenal Scan or MRI | L adrenal mass of 4x4x3.6cm | R adrenal mass of 7.8cm | L adrenal mass of 5.2x4.3cm | R adrenal mass of 55x40x37cm |
| MIBG Scintigraphy Uptake (Y/N) | - | - | - | - |
| Coronary angiography | No CAD | No CAD | No CAD | No CAD |
| Genetic Mutation | - | - | - | - |
| Recurrence (Y/N) | - | - | N | - |
| Emergency adrenalectomy (Y/N) | N | N | N | - |
| Cardiogenic pulmonary edema (Y/N) | Y | - | N | - |
| Mechanical ventilation  (Y/N) | N | N | N | - |
| ECMO (Y/N) | N | N | N | - |
| Death (Y/N) | N | N | N | - |

| Author  year | Trongtorsak, A.  2021 | Yuan,L  2021 | Hideaki K  2021 | Yang, L (our case)  2022 |
| --- | --- | --- | --- | --- |
| Sex/Age | M/50 | M/59 | M/52 | F/27 |
| Trigger | - | - | - | - |
| [Clinical](javascript:;) [manifestation](javascript:;) | CP, dyspnea, nausea, and vomiting | Abdominal pain,  Nausea,vomiting | Cough, dyspnea, lower leg edema | Dyspnea, epigastric pain, diarrhea, headache, diaphoresis |
| Blood pressure | High | High | Low | Low |
| Urinary Catecholamine  ULN | M 5x  NM 15x | Elevated | E 1.5x  NE 17x | - |
| Plasma Catecholamine (ULN) | - | - | E 0.4x  NE 17x | M 2x  NE 23x |
| Increase in Troponin  (Y/N) | Y | Y | Y | Y |
| Initial,echocardiograpy (%) | - | - | 14 | 23 |
| ECG | ST elevation | Normal | - | ST elevation |
| Adrenal Scan or MRI | R adrenal mass of 5.7x6.3x6.7cm | L adrenal mass of 5.3cm | R adrenal mass of 5.5cm | L adrenal mass of 4x5 cm |
| MIBG Scintigraphy Uptake (Y/N) | - | - | Y | - |
| Coronary angiography | No CAD | - | No CAD | - |
| Genetic Mutation | - | - | - | Negative |
| Recurrence (Y/N) | - | N | N | N |
| Emergency adrenalectomy (Y/N) | N | N | N | N |
| Cardiogenic pulmonary edema (Y/N) | Y | N | N | Y |
| Mechanical ventilation  (Y/N) | N | N | N | N |
| ECMO (Y/N) | N | N | N | N |
| Death (Y/N) | N | N | N | N |

**Legend:**

M: Male, F: Female

CP: Chest pain, BP: blood pressure

E: Epinephrine, NE: Norepinephrine, D: Dopamine, M: Metanephrine, NM: Normetanephrine

MRI: Magnetic resonance imaging, R: Right, L: Left, MIBG: Metaiodobenzylguanidine

ECG: Electrocardiogram, RBBB: right bundle-branch,

LVEF: Left ventricular ejection fraction, CAD: Coronary artery disease, Y/N: Yes/No

1. Contadini D, Malagoli A, Binno SM, Villani GQ. A pheochromocytoma-induced Takotsubo syndrome: the importance of multimodality imaging approach. Eur heart j-card img. 2017;18(7):820.

2. Gagnon N, Mansour S, Bitton Y, Bourdeau I. TAKOTSUBO-LIKE CARDIOMYOPATHY IN A LARGE COHORT OF PATIENTS WITH PHEOCHROMOCYTOMA AND PARAGANGLIOMA. Endocr pract. 2017;23(10):1178-92.

3. Gravina M, Casavecchia G, D'Alonzo N, Totaro A, Manuppelli V, Cuculo A, et al. Pheochromocytoma mimicking Takotsubo cardiomyopathy and hypertrophic cardiomyopathy: A cardiac magnetic resonance study. Am j emerg med. 2017;35(2):353-5.

4. Iwase J, Yamanaka M. Sudden onset of pheochromocytoma multisystem crisis at 38 weeks of gestation resulted in intrauterine fetal death: A case report. J obstet gynaecol re. 2017;43(10):1644-8.

5. Nomoto Y, Kawano K, Fujisawa N, Yoshida K, Yamashita T, Makita N, et al. Pheochromocytoma Multisystem Crisis Behaving Like Interstitial Pneumonia: An Autopsy Case. Internal med. 2017;56(2):149-52.

6. Okuno T, Hino M, Kiyama R, Shindo K. [Catecholamine Cardiomyopathy Presenting Inverted-takotsubo Pattern Asynergy]. Masui. 2017;66(4):401-4.

7. Schmidt KH, Herholz T, Rodeck J, Abegunewardene N, Kreitner KF, Münzel T. Pheochromocytoma triggers takotsubo syndrome complicated by cerebral and peripheral embolic events. Eur heart j. 2017;38(19):1522-3.

8. Butt K, Ali S, Sattar Z, Ur Rahman A, Burt JR. Funny Lumps, Flaming Pheo, and a Broken Heart: A Rare Case of Pheochromocytoma. Cureus. 2018;10(11):e3646.

9. Demea AD, Dunca DG, Radu RA, Agoşton-Coldea L. Takotsubo syndrome induced by malignant pheochromocytoma in a patient with type 2 papillary renal cell carcinoma - a case report. Clujul Med. 2018;91(2):242-4.

10. Tafreshi S, Naqvi SY, Thomas S. Extra-adrenal pheochromocytoma presenting as inverse takotsubo-pattern cardiomyopathy treated with surgical resection. BMJ Case Rep. 2018;11(1):null.

11. Takeshita Y, Teramura C, Takamura T. Vanishing of ruptured adrenal mass with takotsubo cardiomyopathy. Endocr j. 2018;65(12):1155-9.

12. Afana M, Panchal RJ, Simon RM, Hejab A, Lahiri SW, Khandelwal AK, et al. Pheochromocytoma-Induced Takotsubo Cardiomyopathy. Tex heart i j. 2019;46(2):124-7.

13. Diaz B, Elkbuli A, Ehrhardt JD, McKenney M, Boneva D, Hai S. Pheochromocytoma-related cardiomyopathy presenting as broken heart syndrome: Case report and literature review. Int J Surg Case Rep. 2019;55(null):7-10.

14. Garla VV, Gosi S, Kanduri S, Lien L. A case of catecholamine-induced cardiomyopathy treated with extracorporeal membrane oxygenation. BMJ Case Rep. 2019;12(9):null.

15. Kiamanesh O, Vu EN, Webber DL, Lau E, Kapeluto JE, Stuart H, et al. Pheochromocytoma-Induced Takotsubo Syndrome Treated With Extracorporeal Membrane Oxygenation: Beware of the Apical Sparing Pattern. JACC Case Rep. 2019;1(2):85-90.

16. Mierke J, Loehn T, Linke A, Ibrahim K. Reverse takotsubo cardiomyopathy- life-threatening symptom of an incidental pheochromocytoma: a case report. Eur Heart J Case Rep. 2019;3(4):1-6.

17. Su Z, Wang Y, Fei H. Takotsubo-Like Cardiomyopathy in Pheochromocytoma. CASE (Phila). 2019;3(4):157-61.

18. Chen M, Zhao T, Chen G, Hu S. A rare long-term undetected pheochromocytoma leading to Takotsubo syndrome in an older male patient: a case report. BMC Endocr Disord. 2020;20(1):93.

19. Dai YL, Zhou J, Lin J, Hu JT, Zhao X. [Recurrent Takotsubo syndrome associated with pheochromocytoma: a case report]. Zhonghua Nei Ke Za Zhi. 2020;59(6):464-7.

20. Sakul NFN, Balabbigari NR, Purewal JK, Barrera C, Roberti R. Reverse Takotsubo Pattern in the Setting of Undiagnosed Pheochromocytoma and Pulmonary Embolism: A Rare Presentation. Am J Case Rep. 2020;21(null):e920231.

21. Sato K, Takahashi J, Amano K, Shimokawa H. A case of recurrent takotsubo-like cardiomyopathy associated with pheochromocytoma exhibiting different patterns of left ventricular wall motion abnormality and coronary vasospasm: a case report. Eur Heart J Case Rep. 2020;4(4):1-5.

22. Sethi P, Chang GV, Gowda SN, Elnair R, Fenner R, Lamfers R. Recurrent Catecholamine-Induced Cardiomyopathy and Hypertensive Emergencies: A presentation of Pheochromocytoma and Related Concerns. S D Med. 2020;73(2):78-80.

23. Spapen J, de Filette J, Lochy S, Spapen H. Acute Heart Failure as a First Presentation of Pheochromocytoma Complicated with "Inverted" Takotsubo Syndrome. Case rep endocrinol. 2020;2020(null):2521046.

24. Yuan S, He T, Yang L, Chu Q, Huang W, Dai H. Basal Takotsubo syndrome induced by pheochromocytoma rupture. Cardiovasc J Afr. 2020;31(null):1-4.

25. Jiang X, Zhang W, Fang Q. Pheochromocytoma-related cardiomyopathy presenting as acute myocardial infarction: A case report. Medicine. 2021;100(11):e24984.

26. Maffè S, Dellavesa P, Paffoni P, Bergamasco L, Arrondini M, Valentini S, et al. Takotsubo syndrome and pheochromocytoma: an insidious combination. Monaldi arch chest d. 2021;91(3):null.

27. Marino G, Michielon A, Musumeci MB, Autore C. Takotsubo syndrome: hyperthyroidism, pheochromocytoma, or both? A case report. Eur Heart J Case Rep. 2021;5(8):ytab270.

28. Martínez A, Gallo-Bernal S, Acosta SC, Calixto CA, Isaza N, Isaza D, et al. Biventricular Takotsubo Cardiomyopathy as the Initial Manifestation of a Pheochromocytoma. CASE (Phila). 2021;5(6):363-7.

29. Trongtorsak A, Chaisidhivej N, Kewcharoen J, Ganokroj P, Torpongpun A. Cardiogenic Shock With Reverse Takotsubo Pattern Secondary to Pheochromocytoma: A Case Report. Cureus. 2021;13(11):e19600.

30. Yuan S, He T, Yang L, Chu Q, Huang W, Dai H. Basal Takotsubo syndrome induced by pheochromocytoma rupture. Cardiovasc J Afr. 2021;32(3):171-4.

31. T K, S S, A A, K G. Pheochromocytoma and Takotsubo’s Cardiomyopathy Requiring Extracorporeal Membrane Support: A Report of Two Cases and Review of Takotsubo’s Cardiomyopathy Subtypes. austinjendocrinoldiabetes. 2022;8(2):null.
